# Catalytic Activity of Alkali Metal Cations for the Chemical Oxygen Reduction Reaction in a Biphasic Liquid System Probed by Scanning Electrochemical Microscopy

**DOI:** 10.1002/chem.202001967

**Published:** 2020-07-23

**Authors:** Shokoufeh Rastgar, Keyla Teixeira Santos, Camilo Andrea Angelucci, Gunther Wittstock

**Affiliations:** ^1^ Carl von Ossietzky University of Oldenburg Chemistry Department 261111 Oldenburg Germany; ^2^ Federal University of ABC Center for Natural and Human Sciences Av. dos Estados 5001 09210-580 Santo André/SP Brazil

**Keywords:** biphasic catalysis, chemical oxygen reduction reaction, electrochemistry, hydrated alkali ions, ion transfer voltammetry, phase-transfer catalysis

## Abstract

Chemical reduction of dioxygen in organic solvents for the production of reactive oxygen species or the concomitant oxidation of organic substrates can be enhanced by the separation of products and educts in biphasic liquid systems. Here, the coupled electron and ion transfer processes is studied as well as reagent fluxes across the liquid|liquid interface for the chemical reduction of dioxygen by decamethylferrocene (DMFc) in a dichloroethane‐based organic electrolyte forming an interface with an aqueous electrolyte containing alkali metal ions. This interface is stabilized at the orifice of a pipette, across which a Galvani potential difference is externally applied and precisely adjusted to enforce the transfer of different alkali metal ions from the aqueous to the organic electrolyte. The oxygen reduction is followed by H_2_O_2_ detection in the aqueous phase close to the interface by a microelectrode of a scanning electrochemical microscope (SECM). The results prove a strong catalytic effect of hydrated alkali metal ions on the formation rate of H_2_O_2_, which varies systematically with the acidity of the transferred alkali metal ions in the organic phase.

## Graphical abstract

**At the interface**: Oxygen reduction by decamethylferrocene close to an interface between an aqueous and organic electrolyte solution is catalyzed by hydrated alkali metal cations in the order K^+^<Na^+^<Li^+^ being transferred from the aqueous to the organic phase by a suitable potential across the liquid|liquid interface. The reaction was followed by amperometric product detection in the aqueous phase.

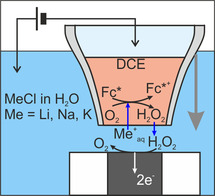

## Introduction

Liquid|liquid interfaces formed between two immiscible electrolyte solutions represent a biomimetic reaction system for advanced oxidation of organic and metallorganic substrates, the formation of reactive oxygen species, and integrated extraction of reaction products.^1^ Although the conduction of the reaction may be as simple as stirring a biphasic liquid mixture and separating the two phases, such systems have much more to offer if they are combined with a control of the transfer processes of ions, electrons, and neutral species across the liquid|liquid interface.

In this realm, ion‐coupled electron transfer reactions such as dioxygen (O_2_) reduction to hydrogen peroxide (H_2_O_2_) and (photo)generation of H_2_ were studied with model compounds as molecular electron donors, including decamethylferrocene (DMFc),1c, 2 ferrocene (Fc),1c, 2c 1,2‐diferrocenylethane,^3^ decamethylosmocene,^4^ and decamethylruthenocene,^5^ dissolved in the organic phase. In the case of osmocene and ruthenocene derivatives, H_2_ was evolved under light exposure from H^+^ supplied in the aqueous phase (aq.). In all studied cases, the reaction proceeds by pumping the H^+^ into the organic phase (o). The ion transfer (IT) could be controlled precisely either by using a potentiostat to supply the free energy of ion transfer from the aqueous to the organic phase or by addition of a phase transfer catalyst, for example, lithium tetrakis(pentafluorophenyl) borate (LiTB), to the acidic aqueous phase. Subsequently, the reduction reaction occurred in the presence of an appropriate electron donor species.

Although the transfer of a hydrophilic ion from the aqueous to the organic phase is a key step in H_2_ evolution in liquid|liquid systems, the oxygen reduction reaction (ORR) by DMFc can occur even in the absence of acidity in the aqueous phase.^6^ It is suggested that instead of a hydrated proton, a solvated alkali metal cation M^+^_(aq.)_ is transferred, which is then able to provide a proton for the reaction according to the following scheme^6^ [Eq. (1), Eq. (2)]:

 

At interface:

(1) $$\left[M\left(H{}_{2}O\right){}_{n}\right]{}^{+}{}_{\left(\mathrm{aq}.\right)}\to \left[\mathrm{MOH}\left(H{}_{2}O\right){}_{n-1}\right]{}_{\left(o\right)}+\left\{H{}^{+}\right\}{}_{\left(o\right)}$$

 

Inside the organic phase:

(2) $$2\;\left\{H{}^{+}\right\}{}_{\left(o\right)}+2\;\mathrm{DMFc}{}_{\left(o\right)}+O{}_{2,(o)}\to H{}_{2}O{}_{2,(o)}+2\;\mathrm{DMFc}{}^{+}{}_{\left(o\right)}$$

This sequence has been demonstrated for Li^+^, in which the hydrophilic cation polarizes the water molecules of its hydration shell, making them acidic in an aprotic polar solvent like dichloroethane (DCE). The slightly acidic water of the hydration shell can then donate protons for both the oxygen reduction and hydrogen evolution reactions with DMFc as electron donor.^6^ In this context, a systematic understanding of the catalytic action of other metal cations as well as a quantitative characterization of their reactivity effects on ion‐coupled electron transfer reactions such as biphasic ORR is of fundamental importance for the evaluation of interfaces in fluidic systems for novel controlled reagent delivery systems, energy‐related systems, or advanced oxidation of organic substrate.1a, 1b, 1d, 1f, 1g, 7 This may also concern reactions at the liquid|liquid interface either to control polymer microstructures,^8^ to study reaction kinetics,^9^ the effects of counter ions and doping,^10^ or to prepare smart nanocarriers such as synthetic polymer shells with an aqueous core.^11^ This also includes the use of phase‐transfer catalysts to conduct the reaction selectively in one phase of choice, for example, to protect the product from hydrolysis in the aqueous phase,^12^ or to design molecular click reactions at a liquid|liquid interface.^13^

Interfacial reaction and mass transport processes have been disentangled by placing an microdisk electrode as a sensor close to any liquid|liquid interfaces and using the instrumentation for scanning electrochemical microscopy (SECM).^14^ Reaction products that were detected by SECM include H_2_O_2_,^15^ O_2_,^16^ H_2_ 15f, 17 in the substrate‐generation/tip collection (SG/TG) mode. Recently, the feedback mode of SECM was used to study chemically polarized liquid|liquid interfaces, that is, systems in which a Galvani potential difference $\Delta_{o}^{w}\varphi$ between the two immiscible electrolyte solutions was formed by partitioning of a common ion. Such systems also facilitate the spontaneous assembly of charged photoactive nanoparticles, for example, BiVO_4_. SECM was used to study the reaction of photogenerated holes and conduction band electrons at nanoparticle‐decorated liquid|liquid interfaces.^16^, ^18^ The surface interrogation mode (SI‐SECM), which can be considered as a transient feedback experiment, was used to assess the amount and the decay kinetics of photogenerated surface‐bound intermediates of the water oxidation reaction at BiVO_4_‐decorated liquid|liquid interfaces.^19^

**Figure 1:**
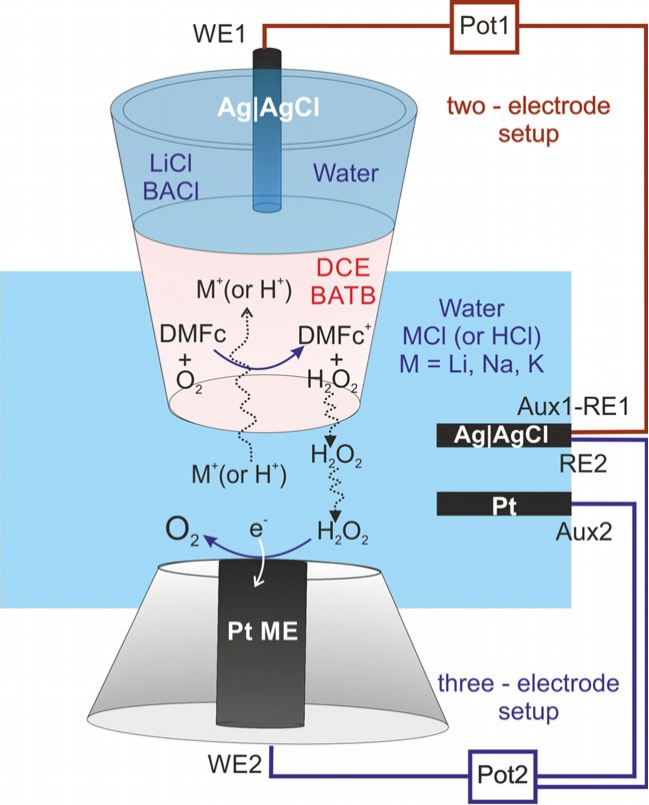
Schematic representation of the liquid|liquid interface at the opening of a micropipette (MP) in close proximity to the Pt microelectrode (ME) operated in the SG/TC mode of SECM.

Here, we use a modified setup for SECM for operando studies of the catalytic behavior of alkali metal ions during oxygen reduction at an externally biased liquid|liquid interface. For that purpose, the liquid|liquid interface under study is mechanically stabilized at the orifice of a micropipette (MP, Figure 1). This setup also enables the application of a well‐defined potential difference across the liquid|liquid interface by a potentiostat in a two‐electrode arrangement between working electrode 1 (WE1) inside the pipette and a combined auxiliary and reference electrode (Aux1‐RE1) in the aqueous solution. This adjustable potential is used to drive the transfer of alkali metal ions across the interface. Here, ion‐transfer cyclic voltammetry (ITCV) is recorded for different ions at the MP with the liquid|liquid interface. The colinearly positioned Pt microelectrode (ME, WE2) is biased by a second potentiostat in a three‐electrode cell and is used for detection of ORR products (e.g., H_2_O_2_) in the SG/TG mode.

## Results and Discussion

### Ion‐transfer cyclic voltammetry

**Figure 2:**
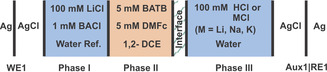
Potentiostatically polarized liquid|liquid interface with the compositions of the two‐electrode electrochemical cells used for ion‐transfer voltammetry.

The composition of the cell comprising an aqueous reference solution, an aqueous phase, and an organic phase is outlined in Figure 2. The interface under study is formed between phases II and III. Phase II contains 5 mm DMFc as reductant and 5 mm of very hydrophobic electrolyte bis(triphenylphosphoranylidene) ammonium tetrakis(pentafluorophenyl)borate (BATB). The aqueous phase III contains 100 mm of either hydrochloric acid (HCl), lithium chloride (LiCl), sodium chloride (NaCl), or potassium chloride (KCl).

**Figure 3:**
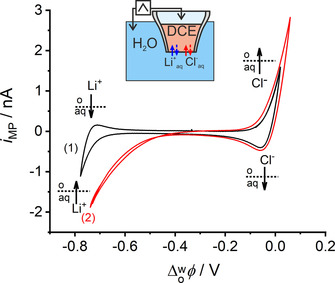
ITCV with 0.1 m LiCl as aqueous electrolyte solution, in the (1) absence and (2) presence of 5 mm DMFc in DCE phase; *r*_MP_≈10 μm and *v*=20 mV s^−1^.

Figure 3 shows the ITCV obtained at the water|DCE interface at the orifice of the MP when using the two‐electrode electrochemical cell outlined in Figure 2. The electrochemical response in Figure 3 curve 1 exhibits a potential window of about 0.8 V for the background electrolytes, that is, Li^+^ and Cl^−^ ions in the aqueous phase and BA^+^ and TB^−^ in DCE. There is an asymmetric diffusion layer for an ion‐transfer on both sides of the liquid|liquid interface. For a reversible IT from inside the pipette to the outside (egress transfer), linear diffusion inside the elongated taper of the MP controls the mass transport, whereas hemispherical diffusion is the limiting process for the ion‐transfer from outside the pipette to the inside (ingress transfer).14e The two diffusion regimes are associated with qualitatively different shapes of the resulting voltammetric curves and allow an assignment of the signals to specific transfer processes, which is usually difficult for macroscopic liquid|liquid interfaces.^20^ The ingress transfer leads to a steady‐state current and the egress transfer results in a peak‐shaped wave. At negative potentials at WE1 inside the MP versus the external Aux1‐RE1, the potential window is limited either by the ingress transfer of Li^+^ to the MP, or by the egress transfer of TB^−^ from the MP. There is a peak at the reverse scan indicating that the current is due to an IT of from inside to the outside of the pipette. Consequently, this side of the potential window is limited by the transfer of Li^+^, as the return peak must correspond to the transfer of an aqueous cation back from the MP after having transferred during the forward scan. The positive end of the potential window is determined by the transfer of either BA^+^ or Cl^−^. A peak current is observed when the potential scan is reversed. This is caused by a linear diffusion process of an ion inside the MP, which has entered the pipette in the forward scan. Thus, this side of the potential window is limited by the transfer of Cl^−^.

**Figure 4:**
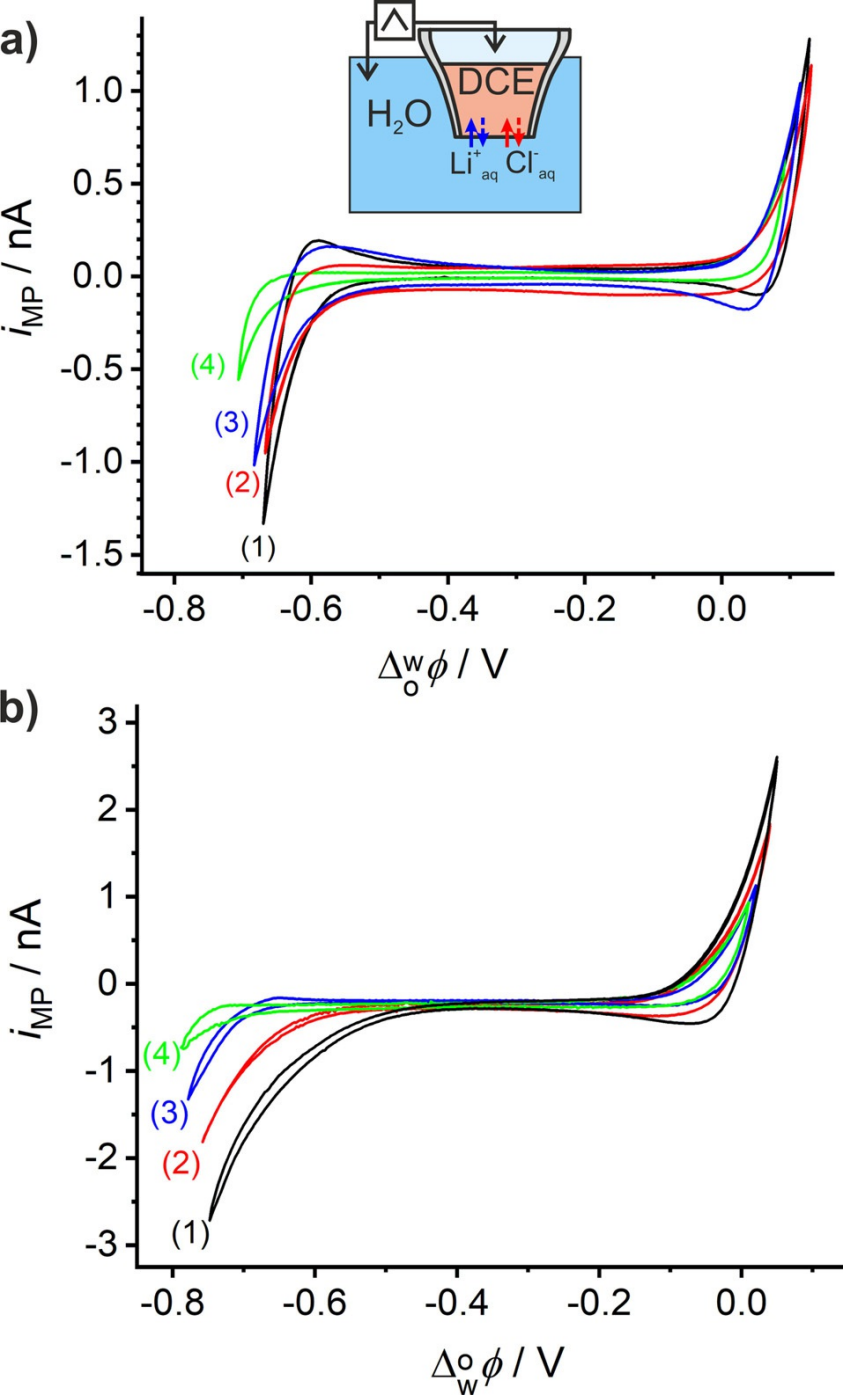
(a) ITCV with 0.1 m of (1) KCl, (2) HCl, (3) LiCl, and (4) NaCl as aqueous electrolyte solution in the absence of DMFc in the organic phase. (b) ITCV with the cell shown in Figure 2 and 0.1 m of (1) KCl, (2) HCl, (3) LiCl, and (4) NaCl as aqueous electrolyte solution in the presence of 5 mm DMFc in the organic phase; *r*_MP_≈10 μm and *v*=20 mV s^−1^.

As shown in Figure 4 a, the current wave at the negative potential limit shifts to different values for Li^+^, Na^+^, and K^+^ and H^+^ for identical initial concentrations of [MCl]=[HCl]=0.1 m for all of the cations in the aqueous phase. Consequently, different potential windows are available in the corresponding electrolytes, which is in good agreement with the tabulated transfer potentials ($\Delta_{o}^{w}\varphi$ ) of those cations that are in the sequence Na^+^>Li^+^>H^+^>K^+^,^21^ unless, the experimental conditions, for example, the concentration of electrolytes, size of liquid|liquid interface, reverse the sequence between Na^+^ and Li^+^.^22^

Upon addition of DMFc as electron donor to the DCE phase, the negative current wave at $\Delta_{o}^{w}\varphi$ =−0.65 V increases for the Li^+^ transfer from the aqueous to the organic phase inside the MP (Figure 3, curve 2). Additionally, the onset of the current wave shifts by approximately 0.2 V towards positive potentials. The peak‐shaped response in curve 1 disappears from the reverse half scan of curve 2. The peak in curve 1 is caused by the back transfer of Li^+^ from the inside of the pipette to the outside. The irreversible transfer of a Li^+^ from the aqueous to the organic phase in the presence of DMFc demonstrates that the solvated Li^+^ ions enter into the ORR in this biphasic system, which may be associated with the production of H_2_O_2_, in agreement with previous reports about the catalytic role of Li^+^ in ORR.^6^ The Li^+^ ion facilitates the transfer of H^+^ needed for ORR. Therefore, its role has been described as that of a phase‐transfer catalyst. The positive current wave for transfer of Cl^−^ from the aqueous to organic phase also increases in the presence of DMFc without change in the peak potential. Furthermore, the peak‐shaped response for the back transfer of Cl^−^ to the aqueous phase does not disappear and does not diminish. This is in strong contrast to the observation for the cation transfer. Therefore, we conclude that Cl^−^ ions do not have any catalytic effect in the presence of DMFc. This is also not expected because Cl^−^ acts as a Lewis base and cannot transfer a H^+^ from a water molecule of the hydration shell to DMFc despite the fact that Cl^−^ can transfer into the organic phase along with its hydration shell (similar but not equal to Li^+^).^23^ The current increase in the both half scans may have the following reasons: ion‐pairing between Cl^−^ and Li^+^ as a result of higher ion‐transfer currents in the presence of DMFc for *both* half cycles. Alternatively, an increased capacitance of the liquid|liquid interface owing to the larger flux of Li^+^ in presence of DMFc could also explain the increased currents in both half cycles.

A similar catalytic behavior was also observed by us in ITCV of HCl and other aqueous alkali chloride solutions such as NaCl and KCl in the presence of DMFc (Figure 4 b). The results demonstrate the catalytic role of alkali cations and H^+^ in ORR in this biphasic system. However, the comparison of the catalytic behavior is not informative or reliable when only using ion‐transfer CV. Accordingly, the SECM setup is developed for the miniaturized liquid|liquid interface as explained below. The biphasic ORR with DMFc as reductant could be demonstrated for the first time with aqueous Li^+^ and in the absence of an excess H^+^ in the aqueous phase by using the capillary methodology and a simple two‐electrode setup. This extends the scope of previous reports in which this reaction was conducted with transfer of H^+^ from the aqueous to the organic phase in a more conventional four‐electrode liquid|liquid electrochemical cell or droplet electrode configuration.2b, 15a

### SECM measurements: in situ electrochemical detection of hydrogen peroxide

**Figure 5:**
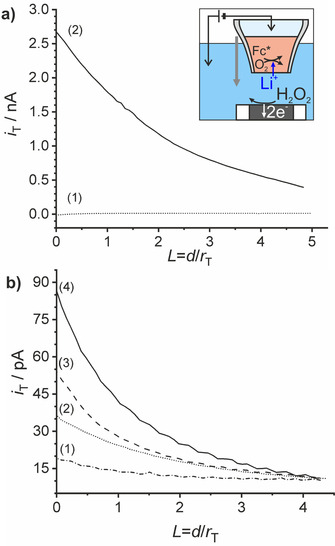
(a) Approach curves (1) without and (2) with $\Delta_{o}^{w}\varphi$ =−0.65 V applied at the liquid|liquid interface using the cell in Figure 2 with LiCl as an aqueous electrolyte solution. (b) The same as (a) but with aqueous electrolyte solutions of (1) HCl, (2) KCl, (3) NaCl, and (4) LiCl; Pt ME with *r*_tip_=12.5 μm as WE2, *E*_T_=0.8 V (vs. Ag|AgCl|Cl^−^), *v*_T_=0.8 μm s^−1^, $\Delta_{o}^{w}\varphi$ =−0.65 V, *r*_MP_≈50 μm for (a) and 10 μm for (b).

To corroborate the ITCV results, we aimed for the direct detection of the ORR reaction product. In a previous report, H_2_O_2_ was detected as a product of O_2_ reduction by DMFc at the liquid|liquid interface with an acidic aqueous solution by using a three‐electrode droplet configuration in a SECM setup.15a The detector for H_2_O_2_ was the positionable ME of the SECM instrument with the specific advantage that H_2_O_2_ is collected before it can be diluted in the aqueous phase. This greatly enhances the sensitivity of the detection method. In our setup, the H_2_O_2_ oxidation current is recorded at the ME, which is colinearly aligned with the MP (Supporting Information, section S3) and moves from the bulk of the aqueous solution towards the liquid|liquid interface while recording the current. The potential applied to the MP drives solvated cations over the interface. Surprisingly, H_2_O_2_ is also detected, and hence ORR proceeds in the absence of a surplus of H^+^ in the aqueous phase (Figure 5 a, curve 2). The ORR can still occur provided that alkali metal cations are transferred from water to the DCE phase, a process that can be externally controlled by applying a suitable negative potential of $\Delta_{o}^{w}\varphi$ =−0.65 V at the liquid|liquid interface. The precise value is obtained for diffusion‐controlled transfer of alkali metal cations by ITCV such as in Figure 4 a. In contrast, the ME current changes only negligibly during the approach to the liquid|liquid interface if no potential drop is applied across the liquid|liquid interface under otherwise identical conditions (Figure 5 a, curve 1). In this situation, no Li^+^ can transfer from the aqueous to the organic phase and hence ORR and H_2_O_2_ production cannot happen.

On approaching the interface under the conditions of ion transfer in Figure 5 a, curve 2, the H_2_O_2_ oxidation current increases because of the higher H_2_O_2_ concentration close to the interface. This marks the liquid|liquid interface as the local source of H_2_O_2_. Additional approach curves in Figure S4 (in the Supporting Information) show negligible oxidation current upon approach of the MP to the ME for liquid|liquid interfaces biased at different potentials within the available potential window from ITCV in Figure 3, curve 1. This confirms the necessity of Li^+^ transfer from the aqueous phase to the organic phase to facilitate the ORR by DMFc in the organic phase.

Figure 5 a proves the dependence of ORR on the presence of Li^+^ transferred from the aqueous to the organic phase. Recently, Girault and co‐workers^6^ proposed a mechanism for this reaction [Eqs. (3), (4), (5)] Briefly, the mechanism considers the hydrophilic alkali ion (e.g., Li^+^_(aq.)_) as Lewis acidic towards water molecules of their solvation shell. When transferred to the organic phase, Li^+^_(aq.)_ can transfer a slightly acidic proton to DMFc, forming [DMFc‐H]^+^. This species can enter into the ORR similarly to solvated protons.2a, 2b

(3) $$\left[\mathrm{Li}\left(H{}_{2}O\right){}_{n}\right]{}^{+}+\mathrm{DMFc}\to \left[\mathrm{LiOH}\left(H{}_{2}O\right){}_{n-1}\right]+\left[\mathrm{DMFc}\text{-}H\right]{}^{+}$$

(4) $$\left[\mathrm{DMFc}\text{-}H\right]{}^{+}+O{}_{2}\to \left[\mathrm{DMFc}\cdot \cdot \cdot H\cdot \cdot \cdot O{}_{2}\right]{}^{+}\to \mathrm{DMFc}{}^{+}+\mathrm{HO}{}_{2}{}^{•}$$

(5) $$\mathrm{HO}{}_{2}{}^{•}+\mathrm{DMFc}+\left[\mathrm{Li}\left(H{}_{2}O\right){}_{n}\right]{}^{+}\to H{}_{2}O{}_{2}+\mathrm{DMFc}{}^{+}+\left[\mathrm{LiOH}\left(H{}_{2}O\right){}_{n-1}\right]$$

**Figure 6:**
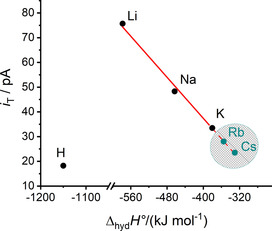
Plot of H_2_O_2_ oxidation at the ME in the cell in Figure 2 for aqueous solutions of LiCl, NaCl, KCl, and HCl as a function of standard hydration enthalpy Δ_hyd_ *H*° of protons and alkali metal ions from ref. 25. The values of *i*_T_ are taken from Figure 5 b at *d*_ME–MP_=2 μm. The solid line is a fit for LiCl, NaCl, and KCl *i*_T_ [pA]=−0.21 Δ_hyd_ *H*° [kJ mol^−1^]−45.70 (*R* ^2^=0.995). The dashed line is an extrapolation of the solid line for RbCl and CsCl solutions and the literature values of Δ_hyd_ *H*°(Rb^+^) and Δ_hyd_ *H*°(Cs^+^).

All the above reactions take place in the DCE phase. However, owing to the presence of the liquid|liquid interface, LiOH and H_2_O_2_ will transfer into the aqueous phase. Under these conditions, the calculated total Gibbs free energies for the reaction [Eqs. (3)–(5)] are −111, −108, −96 kJ mol^−1^ for Li^+^, Na^+^, and K^+^, respectively, which are clearly thermodynamically favorable (Supporting Information, section S5). However, the Gibbs free energy in the absence of M^+^ is +57.3.6a In this comparison, the term $\Delta_{DCE}^{w}G_{M^{+}}^{o,w\to DCE}$ for the back transfer of the alkali metal cation (as an ion‐pair with OH^−^) makes the energetic difference that favors the chemical ORR after the enforced transfer of alkali metal ions. The differentiation between the cations is in line with the solvation of those cations according to the non‐Bornian solvation model taking into account the charge, hydration radius, and hydration number of each cation.23a, 23b, 23c A similar sequence is reached by looking at the hydration enthalpies (Figure 6) and the hydration entropy, which make only a small modification to the trend from the enthalpies (Δ*S* ${}_{\mathrm{Li}{}^{+}}$ =−142 J mol^−1^ K^−1^, Δ*S* ${}_{\mathrm{Na}{}^{+}}$ =−103 J mol^−1^ K^−1^, Δ*S* ${}_{K{}^{+}}$ =−88 J mol^−1^ K^−1^).^24^ These calculations confirm that the presence of M^+^ is essential for such reactions to proceed.

The procedure was also applied to other alkali metal cations to compare their catalytic activity for biphasic ORR. Figure 5 b shows a comparison of the SG/TC approach curves based on H_2_O_2_ oxidation at the ME for different aqueous alkali metal chloride solutions. The oxidation currents increase with a clear trend: KCl<NaCl<LiCl. The different approach curves must originate from different H_2_O_2_ generation rates caused by the presence of the different solvated alkali metal cations transferred to the organic phase. This sequence of K^+^<Na^+^<Li^+^ measured for the different electrolyte solutions at a particular distance is in excellent agreement with the sequence of their acidity and standard hydration enthalpy Δ_hyd_ *H*°.^25^ Figure 6 shows quantitatively the relationship between the H_2_O_2_ oxidation current at the ME at a distance *d*_ME–MP_=2 μm between the ME and the MP (from Figure 5 b) and Δ_hyd_ *H*° from the literature.^25^ The position at which *d*_ME–MP_=0 is evident when ME and MP touch each other and the ME current changes abruptly. The H_2_O_2_ oxidation current is an indicator of the ORR rate in the organic phase. Consequently, the observation confirms the role of the hydration shell of alkali metal ions and their surprising acidity within the mechanism for catalytic reduction reaction of O_2_ by DMFc in DCE [Eq. (3)]. The observed linear correlation for values of LiCl, NaCl, and KCl allows a prediction for the rate of ORR when Rb^+^ or Cs^+^ ions are to be transferred. A linear extrapolation yields an expectation of 28 pA for Rb^+^ and 22 pA for Cs^+^.

Depending on the hydration shell surrounding the cations,^23^ different ORR catalytic modes of action could be expected for cations in the organic phase. Cations of high charge density interact more strongly with the negative charge centers of water (oxygen atoms) in their hydration shells. This results in higher acidity and hence stronger facilitation of DMFc‐H^+^ formation as the first step in the ORR. However, hydrophobic and semihydrophobic cations strip the hydration shell upon transfer and are solvated predominately by DCE molecules in the organic phase.^23^ Hence, their ORR activity is expected to be below those of the alkali metal ions.

Interestingly, the current observed in the presence of HCl in the aqueous phase (Figure 5 b, curve 1) is much smaller than those of the tested alkali metal chloride solutions. Several effects may contribute to this phenomenon. The equilibrium concentration is 0.1 m H_2_O dissolved in DCE in a H_2_O|DCE biphasic system.^26^ In the case of the HCl aqueous electrolyte, the transferred protons are consumed, leaving behind an unbalanced Cl^−^ excess in the aqueous phase. In case of MCl, the transfer of M^+^ and subsequent reaction leads to an alkalization of the organic phase. M^+^ can be transferred back to the aqueous phase, restoring the initially stable electrolyte and counteracting the Cl^−^ excess. In such a situation also [DMFc^+^][OH^−^] could transfer to the aqueous phase in the presence of the produced OH^−^ in the organic phase ($\Delta_{DCE}^{w}G_{{DMFc}^{+}}^{o,w\to DCE}$ =24.1 kJ mol^−1[6a]^). This may facilitate an ongoing reaction. However, owing to the multitude of possible transfer processes and associated free energy contributions, it is very difficult with the available techniques to completely disentangle the different effects.

### Chronoamperometry measurements: quantitative detection of hydrogen peroxide

**Figure 7:**
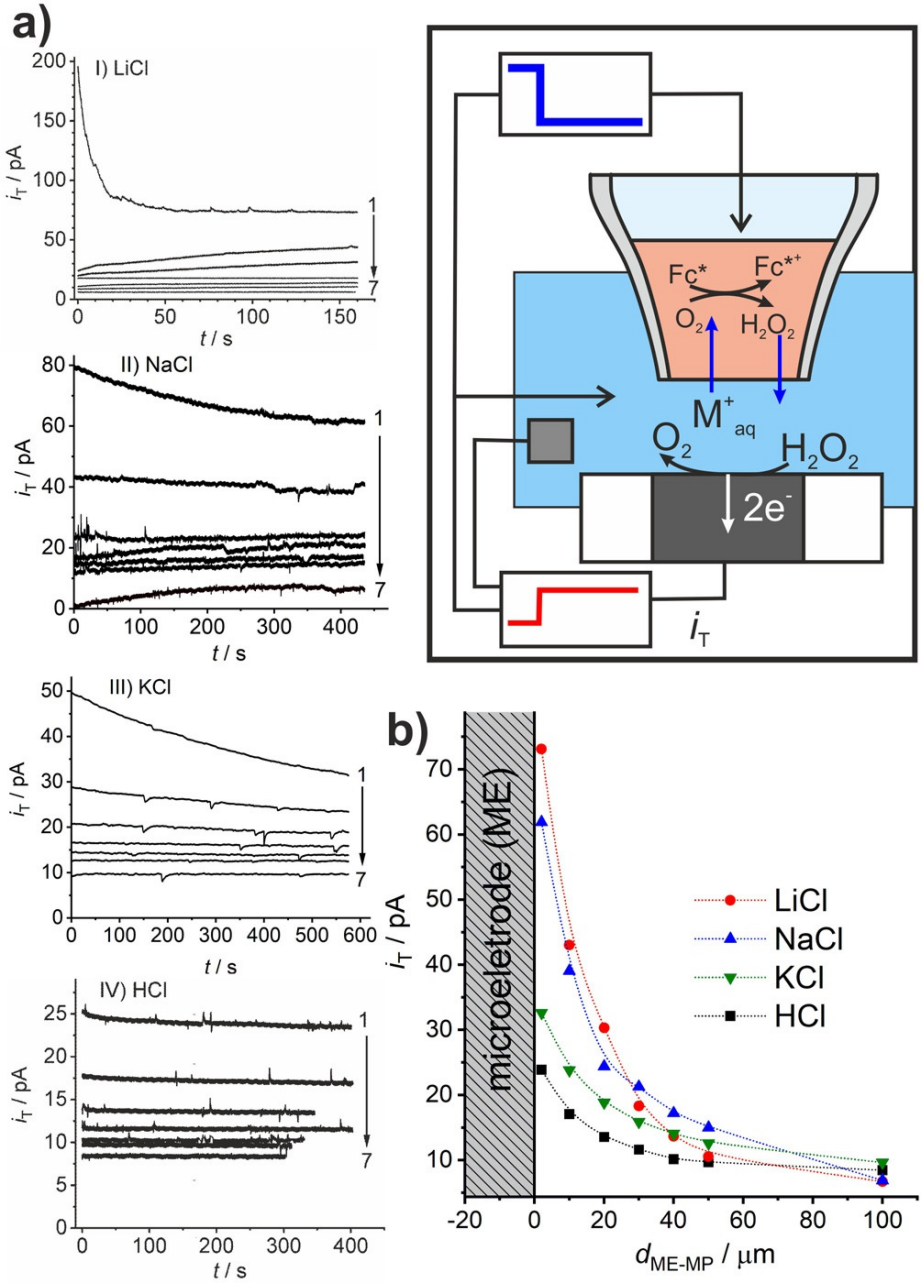
(a) Chronoamperograms for the H_2_O_2_ oxidation at the ME after ion‐transfer and H_2_O_2_ generation at the liquid|liquid interface by using the cell in Figure 2; the aqueous solution was (I) LiCl, (II) NaCl, (III) KCl, and (IV) HCl at *d*_ME–MP_ values of (1) 2, (2) 10, (3) 20, (4) 30, (5) 40, (6) 50, and (7) 100 μm. (b) Steady‐state oxidation current from the chronoamperograms in (a) as a function of *d*_ME–MP_ for different aqueous electrolytes; Pt ME with *r*_T_=12.5 μm, *E*_T_=0.8 V (vs. Ag|AgCl|Cl^−^), $\Delta_{o}^{w}\varphi$ =−0.65 V, and *r*_MP_=10 μm.

During the recording of an approach curve, the diffusion layer above the MP may not attain a complete steady state but expand slightly during the experiment. A more defined situation is obtained by chronoamperometry, in which the ME and MP are kept at a fixed distance *d*_ME–MP_ and the potential of the MP is changed at the start time *t*_0_=0 from a value at which no ion transfer occurs, to the potential $\Delta_{o}^{w}\varphi$ =−0.65 V, which causes ion transfer from the aqueous to the organic phase (Figure 4 a). At the same time, the ME potential *E*_T_ is changed from 0 V to +0.8 V (vs. Ag|AgCl|Cl^−^) to detect H_2_O_2_ by oxidation over several hundred seconds. Afterwards, the potentials are switched back to stop the reactions. After a waiting period of 70 s to allow the diffusion layers to relax, *d*_ME–MP_ is incremented and the procedure is repeated. The potential jump at the MP defines an exact onset for the ion transfer across the liquid|liquid interface relative to the current measurement at the ME. Figure 7 a shows the chronoamperometric transients at the ME in the four electrolytes and at different distances *d*_ME–MP_ between 2 μm to 100 μm. The steady‐state currents of each transient are plotted together in Figure 7 b for different *d*_ME–MP_. The oxidation current for each alkali chloride electrolyte solution increases as *d*_ME–MP_ decreases. This behavior is in accordance with the approach curves in Figure 5 b, which capture a condition very close to the steady‐state current. It also reproduces the sequence of catalytic activity of the three tested alkali metal ions in Figure 5 b. It thus corroborates the proposed mechanism for catalytic ORR in the presence of those hydrated cations and DMFc in the organic DCE phase [Eqs. (3)–(5)].

Interestingly, there are qualitative differences in the current transients both at different distances and between different aqueous electrolytes. For LiCl and the smallest distance, the transient starts with the highest currents and decays to the steady‐state value. In this case, the collection efficiency is very high and the transition time between the onset of the reaction and product detection at the ME is not resolved. The current decays owing to the fast depletion of oxygen in the reaction zone in the DCE phase. The steady‐state current is then controlled by the mass transport of O_2_ into the reaction zone. At larger distances, the collection efficiency of the ME gradually decreases, thus decreasing the steady‐state currents. Also, the transients rise more steadily because there is a considerable time delay between the onset of the reaction and the detection at the microelectrode. For NaCl, the same trend is observed with a slight modification. For very short distances, the initially recorded current is lower than for LiCl electrolyte but the transient decays slower to the steady‐state values. Because Na^+^_(aq.)_ is less active than Li^+^_(aq.)_, the initial reaction rate is slower and it takes a longer time until the mass transport of O_2_ becomes a significant limitation. For KCl and HCl, the current transients are always decaying. The ORR reaction rate is slower so that other limiting factors are not important.

Please note that the order of reactivity H^+^<K^+^<Na^+^<Li^+^ does not follow the sequence of the onset of IT in Figure 4 a. The highest voltage is required for transfer of Na^+^ followed by Li^+^, H^+^, and K^+^. If it were just the magnitude of cation transfer to the DCE phase caused by the applied potential of $\Delta_{o}^{w}\varphi$ =−0.65 V during the experiments, the sequence of H_2_O_2_ production rates should follow the order NaCl<LiCl<HCl<KCl, which is qualitatively different from our experimental observation. This adds strong evidence that the acidity of the hydrated cations in the organic phase is indeed the decisive factor for the observed behavior.

## Conclusion

The oxygen reduction reaction using DMFc as the electron donor at a water|DCE interface represents an example of ion‐coupled electron transfer reactions at a liquid|liquid interface. The reaction can proceed by catalysis of hydrated alkali metal ions transferred from the aqueous to the organic DCE phase. Ion‐transfer cyclic voltammetry demonstrates the transfer of H^+^, Li^+^, Na^+^, and K^+^ across the water|DCE interface if the externally applied Galvani potential difference exceeds the ion‐specific value of the transfer potential $\Delta_{o}^{w}\varphi$ . To compare the catalytic activity of different alkali metal ions, the coupling of the micropipette and an amperometric microelectrode is demonstrated. The MP mechanically stabilizes the liquid|liquid interface, whereas the ME is used for product detection in the substrate‐generation/tip collection mode of SECM. With this combination, the catalytic action of the transferred hydrated alkali metal ions was compared. The activity increases in the sequence K^+^<Na^+^<Li^+^ in strong correlation with literature values for the standard hydration enthalpies of those ions. It demonstrates the catalytic biphasic ORR for H_2_O_2_ formation in the presence of different alkali metal cations in aqueous electrolyte solutions. Accordingly, the cations with higher hydration enthalpy can induce a higher driving force for ORR and, therefore, higher rate for H_2_O_2_ generation. Interestingly, transferred hydrated protons do not accelerate the ORR to the same extent as alkali ions, probably as a result of the higher energy of the resulting water cluster in DCE.

Taken together, the demonstrated methodology provides very useful combined information for ion and electron transfer reactions, which is a prerequisite for optimizing biphasic fluidic systems with respect to the composition of the aqueous and organic phases. This also will allow us to generalize the chosen example of H_2_O_2_ generation by utilization of a wider range of electron donors and oxidants species with the ultimate goal of carrying out vital ion‐coupled electron transfer reactions in energy‐related or synthetic fluidic systems in which a heterogeneous liquid system facilitates the work‐up and regeneration of the solutions. This also refers to organic synthetic protocols “on water”,^27^ in which the amount of organic solvents can be reduced. This is interesting for the reduction of organic waste but also for the synthesis of water‐soluble materials, for which optimized biphasic systems can be advantageous in terms of improved kinetics, higher selectivity, and higher yields.

## Experimental Section

### Chemicals

NaCl (>99.99 %, Carl Roth), LiCl (>9999 %, VWN chemicals), KCl (>99.99 %, Carl Roth), HCl (35 %, VWN chemicals), DMFc (Sigma–Aldrich), LiTB (Sigma–Aldrich), bis(triphenylphosphoranylidene) ammonium chloride (BACl, Sigma–Aldrich), and DCE (Sigma–Aldrich) were used as received. BATB was prepared by metathesis of BACl and LiTB in a molar ratio of 1:1 and recrystallized in a mixture of 2:1 methanol/water before use as the supporting electrolyte in the DCE phase of the biphasic liquid system.^28^

### Preparation of microelectrode and micropipette

The Pt ME was fabricated and polished as described elsewhere.^29^ In short, a Pt wire (25 μm diameter, Goodfellow, Cambridge, UK) was sealed in a borosilicate capillary (outer diameter (O.D.)/inner diameter (I.D.)=1.0 mm/0.5 mm, 100 mm length) under vacuum. After connection to a Cu wire by using silver‐epoxy glue (EPO‐TEK, John P. Kummer GmbH, Augsburg, Germany) and heat treatment inside an oven at 60 °C for 10 h, the ME was polished and shaped into a cone by a wheel with 180‐grid Carbimet paper disks, then polished sequentially with 0.3 μm and 0.05 μm alumina powder on a micropolishing cloth (Buehler, Lake Bluff, IL, USA) for 5 min, interrupted by rinsing with water after each polishing step. MPs with *r*_MP_≤50 μm were fabricated from quartz capillaries (O.D./I.D, 1.0/0.7 mm, Sutter Instrument, Navato, CA, USA) by using a laser puller (P2000, Sutter Instrument) and by adjusting the pulling parameters (heat, filament, velocity, delay, pull) to obtain MPs with short tapers. These MPs yield undistorted voltammograms owing to the small *iR* drop. The inner wall of the pipette was silanized by inserting a small syringe from the back of the pipette and placing a small drop of trimethylchlorosilane close to the end of the pipette. The solution was removed from the pipette after 30 min by a syringe, and the silanized pipette was allowed to dry in the air over 8 h. Silanizing allows the filling of the pipette with the organic electrolyte solution and the formation of the liquid|liquid interface with the outside aqueous electrolyte at the orifice of the pipette.

### Apparatus and procedure

The ITCV and SECM approach curves were obtained by utilizing a home‐built instrument^30^ operated under SECMx.^31^ The SECM setup designed for the study of the liquid|liquid interface at the MP is shown in Figure S1 a (in the Supporting Information). The ME was mounted by means of a chromatography fitting to the bottom of the SECM cell body made from polytetrafluoroethylene. The ME (bottom, facing up) and MP (top, facing down) were aligned to each other as shown in Figure S1 (in the Supporting Information), by moving the MP in the *x*−*y*−*z* directions with a piezoelectric motor (Scientific Precision Instruments, Oppenheim, Germany, detailed in section S3 in the Supporting Information). To avoid crashing, the process was monitored with two cameras whose optical axes were at a right angle to each and parallel to the surface of the ME. The body of the electrochemical cell had walls made from microscopic slides to allow undisturbed optical observation of the relative position of the MP and ME inside the electrochemical cell in *x* and *y* directions. After the initial positioning, the relative *x* and *y* positions between the MP and the ME were fixed during the SECM experiments.

The composition of the aqueous reference solution, the aqueous and the organic phases is outlined in Figure 2. The silanized MP was partially filled with 5 mm DMFc as an electron donor agent and 5 mm BATB as a supporting electrolyte in DCE as solvent. The remaining part of the MP was back‐filled by the aqueous 1 mm BACl and 10 mm LiCl reference solution. The solution on each side of the liquid junction inside the MP (phases I and II) shared BA^+^ as common ion. The aqueous reference solution contained LiCl for establishing the potential at the Ag|AgCl|Cl^−^ electrode used to polarize the liquid|liquid interface (II/III) under study (WE1). The filled MP was immersed in the aqueous phase containing 100 mm HCl or MCl (M=Li, Na, K). Another Ag|AgCl|Cl^−^ electrode immersed in the outer aqueous solution served as Aux1|RE1. The reaction under study (ORR) occurs at or close to the liquid|liquid interface (II/III) formed at the opening of the pipette, which is externally polarized by a two‐electrode setup (Figure 1) and is used for recording ITCV. The ionic current resulting from the transfer of cations from the aqueous to DCE phase (at negative potential) is defined as a negative current.

Simultaneously, a three‐electrode configuration was integrated into the setup described above for recording cyclic voltammograms, chronoamperograms, and SECM approach curves for the detection of H_2_O_2_ as a product of ORR. A Pt ME, a Ag|AgCl|Cl^−^, and a Pt wire in the aqueous phase served as WE2, RE2, and Aux2, respectively. The Pt ME (*r*_T_=12.5 μm) was biased at *E*_T_=+0.8 V vs. Ag|AgCl|Cl^−^ for diffusion‐controlled oxidation of H_2_O_2_ (Figure S2 in the Supporting Information). The SECM approach curves were obtained by moving the biased MP (supporting the liquid|liquid interface, $\Delta_{o}^{w}\varphi$ =−0.65 V for transfer of alkali metal ions) toward the biased ME and recording simultaneously the H_2_O_2_ oxidation current at the ME as a function of *d*_ME–MP_. The approach curves were repeated at different $\Delta_{o}^{w}\varphi$ applied at the liquid|liquid interface with the ME at *E*_T_=+0.8 V vs. Ag|AgCl|Cl^−^. For recording chronoamperograms, the biased Pt ME was kept at fixed distances from the MP (*d*_ME–MP_=2–100 μm) for detection of H_2_O_2_, whereas the liquid|liquid interface was biased at $\Delta_{o}^{w}\varphi$ =−0.65 V.

## Conflict of interest

The authors declare no conflict of interest.

## Supporting information

As a service to our authors and readers, this journal provides supporting information supplied by the authors. Such materials are peer reviewed and may be re‐organized for online delivery, but are not copy‐edited or typeset. Technical support issues arising from supporting information (other than missing files) should be addressed to the authors.

Supplementary
