# Supplementary material for: Catalytic Activity of Alkali Metal Cations for the Chemical Oxygen Reduction Reaction in a Biphasic Liquid System Probed by Scanning Electrochemical Microscopy
Source: Chemistry. 2020 Jul 23;26(47):10882–90. doi: 10.1002/chem.202001967 (PMC7496973; doi:10.1002/chem.202001967)
Supplement: Supplementary file 1 — Supplementary [file CHEM-26-10882-s001.pdf]

Chemistry–A European Journal

Supporting Information

Catalytic Activity of Alkali Metal Cations for the Chemical Oxygen Reduction Reaction in a Biphasic Liquid System Probed by Scanning Electrochemical Microscopy

Shokoufeh Rastgar+,[a] Keyla Teixeira Santos+,[a, b] Camilo Andrea Angelucci,[b] and Gunther Wittstock*[a]

Catalytic activity of alkali metal cations for chemical oxygen reduction reaction in a biphasic liquid system probed by scanning electrochemical microscopy

Shokoufeh Rastgar, Keyla Teixeira Santos, Camilo Andrea Angelucci, Gunther Wittstock*

Table of Contents

S1. The setup for the coupling of SECM and the liquid|liquid interface

S2. Electrochemical oxidation of H_2O_2 at microelectrode

S3. Positioning of the micropipette over the microelectrode in *x*–*y*–*z* directions

S4. SECM approach curve measurements based on H_2O_2 oxidation

S5. Thermodynamic calculation for two-electron oxygen reduction by DMFc in the organic phase in the presence and absence of M^+

S1. The setup for the coupling of SECM and the liquid|liquid interface

The setup designed for the coupling SECM to the liquid|liquid interface is shown in Figure S1a. It includes the electrochemical cell with counter and reference electrodes, the micropipette with a Ag wire covered by AgCl inside the micropipette (as a working electrode, WE1, top), and microelectrode (as a WE2, bottom) facing each other. The position of aligned micropipette relative to the electrode, is shown in Figure S1b,c. Two video microscopes are positioned in the front and side of the cell (*x*-*y* direction).

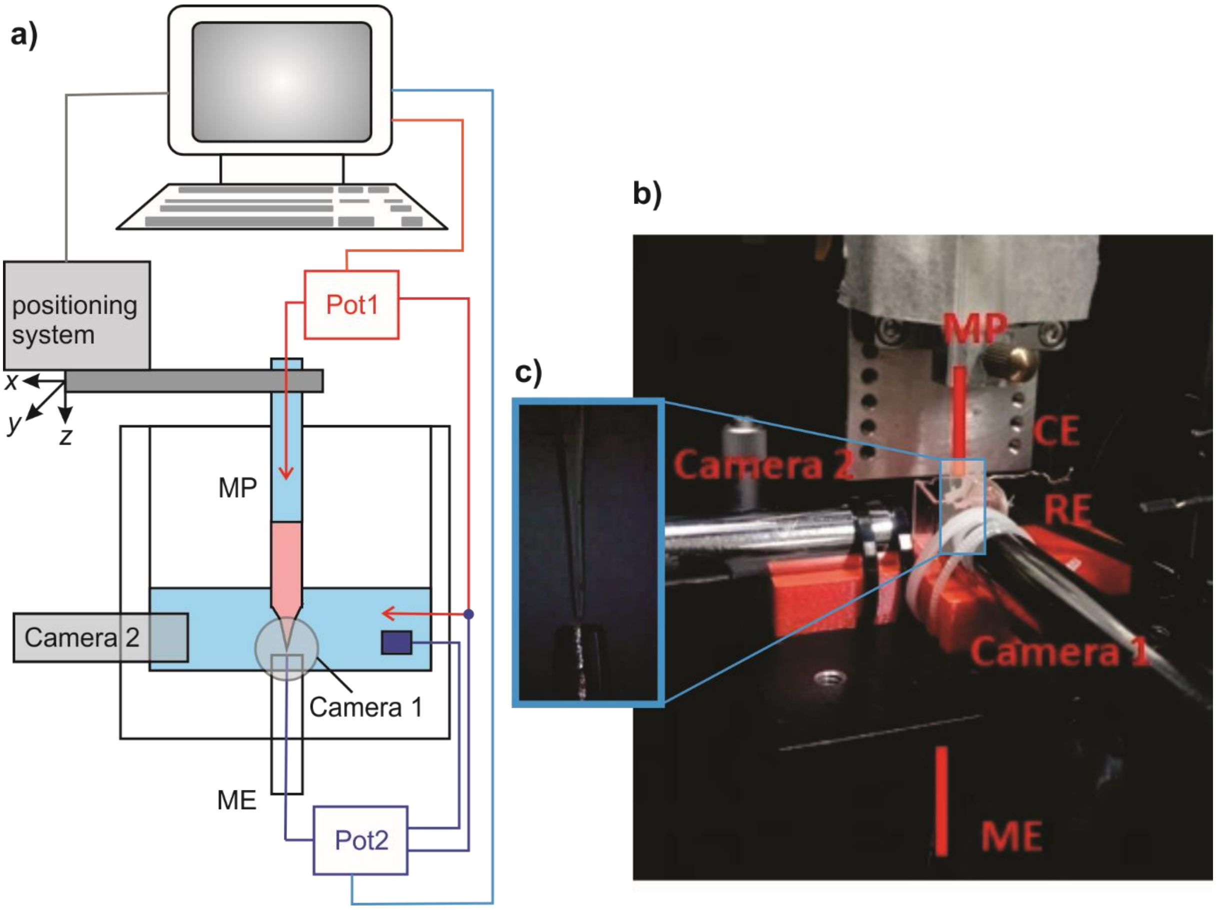

Figure S1. Experimental setup. a) Schematic; b) photographs of the setup of the with the MP and ME and two cameras; c) camera image of the the micropipette (r_{MP} = 10 µm) containing the organic phase and the aligned and positioned Pt microelectrode (r_T = 12.5 µm).

S2. Electrochemical oxidation of H_2O_2 at microelectrode

Cyclic voltammetry is shown in Figure S2 for an aqueous 5 mM H_2O_2 solution with 100 mM LiCl at the Pt ME. Before recording the voltammogram, a potential scan from +0.6 V to -0.6 V at 50 mV s^{-1} was applied to the Pt ME in order to reduce the Pt oxide layer and obtain an active surface. An oxidation peak at 0.8 V appears due to H_2O_2 oxidation. For recording the SECM approach curves based on H_2O_2 oxidation current, a potential E_T = 0.8 V (*vs.* Ag|AgCl|Cl^-) is selected for steady-state, diffusion-controlled H_2O_2 oxidation at the Pt ME.

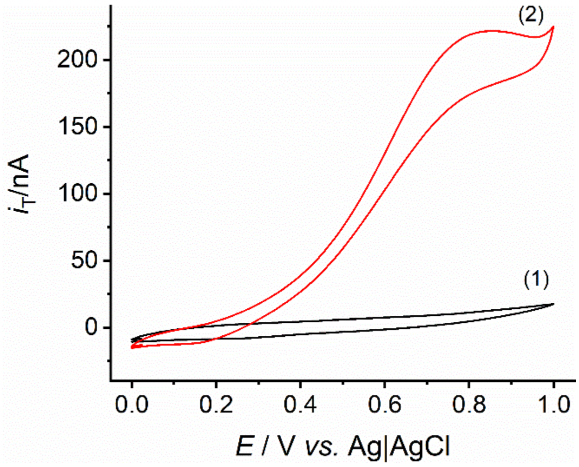

Figure S2. Cyclic voltammetry of (2) 5 mM H_2O_2 in 100 mM LiCl aqueous electrolyte solution; curve (1) shows the blank experiment in 100 mM aqueous LiCl solution at a Pt ME, r_T = 12.5 µm, v = 50 mV s^{-1}.

S3. Positioning of the micropipette over the microelectrode in *x–y–z* directions

The alignment of the MP relative to the ME started by placing the MP 30–100 µm above the ME under the control of the video camera. The MP was biased at $\Delta_o^w\phi$ = -0.65 V to cause the IT. The ME biased at potential of diffusion-controlled oxidation of H_2O_2 in 100 mM LiCl. Then, the MP was moved in *x* direction above the biased ME while monitored the ME current. The oxidation current increased when the MP was placed above the ME, generating H_2O_2 as a result of biphasic ORR (Figure S3a). Then, the MP was held at the position of maximum oxidation current along the *x* direction. From there, the procedure was repeated for the *y* direction in the same way (Figure S3a). Afterwards, the approach curve in vertical (*z*) direction was measured (Figure S3b). Afterwards the MP was placed at a distance $d_{\text{ME-MP}}$ = 2.0 µm. In this situation, the opening of the pipette (representing the liquid|liquid interface) is placed completely above the active ME area as shown in Figure S1c for MP with radius of 10 µm. By overlaying the line scans in *x* and *y* direction, the overlapped area represents the area of the opening of the pipette facing the ME (Figure S3a).

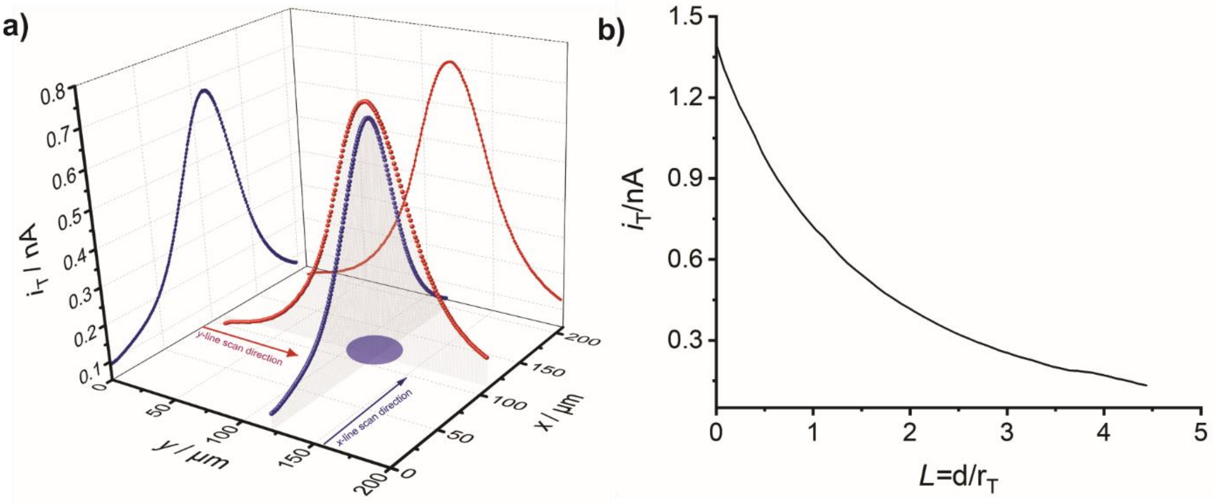

Figure S3. Alignment of MP and ME by line scans based on H_2O_2 oxidation. (a) Overlays of line scans in both *x* and *y* directions. *x*-line scan (blue curve) and then, *y*-line scan (red curve) at the *x*-position of the maximum of the blue curve. (b) SECM approach curve at the (*x*, *y*)-coordinate with the maximum oxidation current. Organic phase is 5 mM DMFc + 5 mM BATB in DCE; aqueous phase is 100 mM LiCl; reference aqueous phase 10 mM LiCl + 1 mM BACl; Pt ME r_T = 12.5 µm, v_T = 0.8 µm s^{-1} in *z* direction and 5 µm s^{-1} in *x* and *y* direction, E_T = 0.8 V (vs. AgCl) for oxidation of H_2O_2, $\Delta_o^w\phi$ = -0.65 V, r_{MP} = 50 µm.

S4. SECM approach curve measurements based on H_2O_2 oxidation

Figure S4 shows the approach curves for H_2O_2 oxidation at the Pt ME towards the liquid|liquid interfaces biased at different potentials $\Delta_o^w \phi$ of 0.05 V, -0.3 V and -0.45 V. All those approach curves show negligible H_2O_2 oxidation currents because no cations can be transferred from the aqueous to the DCE phase at those potentials and hence ORR does not commence. For comparison, the inset of Figure S4 shows the H_2O_2 oxidation current for liquid|liquid interface biased at $\Delta_o^w \phi$ = -0.65 V. The dependence of the H_2O_2 oxidation current at the ME on the Li transfer confirms the necessity of hydrated Li^+ ions for the catalytic biphasic ORR in the presence of DMFc in organic phase.

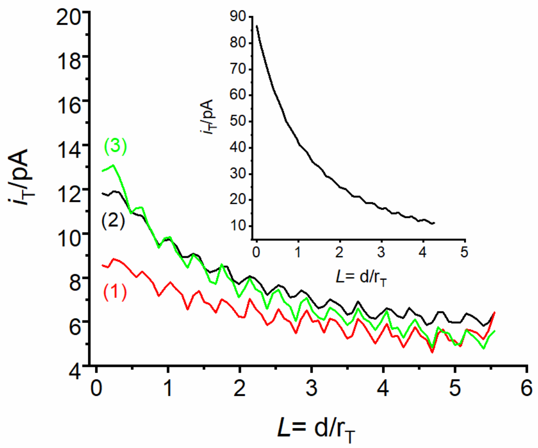

Figure S4. Approach curves with different $\Delta_o^w \phi$ at (1) 0.05 V, (2) -0.45 V and (3) -0.3 V applied at the liquid|liquid interface using the cell in Figure 2 of the main text with aqueous LiCl solution. For comparison, the inset shows the approach curve with $\Delta_o^w \phi$ = -0.65 V applied at the liquid|liquid interface; r_T = 12.5 µm, r_{MP} = 10 µm, E_T = 0.8 V (vs. AgCl) and v_T = 0.8 µm s^{-1}.

S5. Thermodynamic calculation for two-electron oxygen reduction by DMFc in the organic phase in the presence and absence of M^+

The overall reaction comprises and ion-transfer step and the ORR in the organic phase which can be considered as a chemical follow-up reaction of the enforced ion-transfer. As we will see below, the chemical step may again transfer processes across the interface. However, this is restricted to neutral species because the applied potential drives cations from the aqueous to the organic phase. In order to compare the driving forces for the chemical ORR at liquid|liquid interface, the Gibbs free energy $\Delta G^{\circ}_{\text{chem}}$ was calculated for the chemical steps *i*) with alkali metal ions in the organic phase [eq. (S1)] and *ii*) for ORR without alkali metal ions in the organic phase, which is followed by transfer of $[DMFc^+][OH^-]$ ion-pair to the aqueous phase [eq. (S2)].

$$O_{2,(o)} + 2DMFc_{(o)} + 2\,[M(H_2O)_n]^+_{(o)} \rightarrow H_2O_{2,(aq)} + 2DMFc^+_{(o)} + 2\,[M(OH)(H_2O)_{n-1}]_{(aq)} \qquad (S1)$$

$$O_{2,(o)} + 2DMFc_{(o)} + 2\,H_2O_{(o)} \rightarrow H_2O_{2,(aq)} + 2DMFc^+_{(aq)} + 2\,OH^-_{(aq)} \qquad (S2)$$

$\Delta G^{\circ}_{\text{chem}}$ for the M^+-catalyzed path in eq. (S1) can be determined by

$$\Delta G^{\circ}_{\text{chem}} = \Delta G^{\circ}_{\text{et}} - \Delta^{\text{w}}_{\text{DCE}} G^{\circ,\text{w}\rightarrow\text{DCE}}_{H_2O_2} - 2\Delta^{\text{w}}_{\text{DCE}} G^{\circ,\text{w}\rightarrow\text{DCE}}_{M^+} - 2\Delta^{\text{w}}_{\text{DCE}} G^{\circ,\text{w}\rightarrow\text{DCE}}_{OH^-}. \qquad (S3)$$

Here $\Delta G^{\circ}_{\text{et}}$ is the standard Gibbs free energy change of electron transfer and $\Delta^{\text{w}}_{\text{DCE}} G^{\circ,\text{w}\rightarrow\text{DCE}}_{H_2O_2}$, $\Delta^{\text{w}}_{\text{DCE}} G^{\circ,\text{w}\rightarrow\text{DCE}}_{M^+}$ and $\Delta^{\text{w}}_{\text{DCE}} G^{\circ,\text{w}\rightarrow\text{DCE}}_{OH^-}$ are the standard Gibbs free energy of transfer of H_2O_2, M^+ and OH^- between the two phases. We assume that LiOH does not precipitate in organic phase but is quickly extracted to aqueous phase as a $[M^+][OH^-]$ ion-pair. Otherwise, we should consider the extra term $\Delta G^{\text{DCE}}_{\text{sol,M(OH)}}$ for the Gibbs free energy of solvation of MOH in DCE. This is in the direction of disfavoring the whole the reaction.

In the absence of M^+ in the aqueous phase [eq. (S2)], $\Delta G^{\circ}_{\text{tot}}$ is obtained by

$$\Delta G^{\circ}_{\text{tot}} = \Delta G^{\circ}_{\text{et}} - \Delta^{\text{w}}_{\text{DCE}} G^{\circ,\text{w}\rightarrow\text{DCE}}_{H_2O_2} - 2\Delta^{\text{w}}_{\text{DCE}} G^{\circ,\text{w}\rightarrow\text{DCE}}_{DMFc^+} - 2\Delta^{\text{w}}_{\text{DCE}} G^{\circ,\text{w}\rightarrow\text{DCE}}_{OH^-} \qquad (S4)$$

Here, the transfer of $[DMFc^+][OH^-]$ $\Delta^{\text{w}}_{\text{DCE}} G^{\circ,\text{w}\rightarrow\text{DCE}}_{DMFc^+}$ from organic phase to the aqueous phase should be taken into account.

The contribution $\Delta G^{\circ}_{\text{et}}$ is obtained from

$$\Delta G^{\circ}_{\text{et}} = -2F\left(\left[E^{\circ}_{O_2/H_2O_2,OH^-}\right]^{\text{DCE}}_{\text{SHE}} - \left[E^{\circ}_{\text{DMFc/DMFc}^+}\right]^{\text{DCE}}_{\text{SHE}}\right). \qquad (S5)$$

The results are summarized in Table S1. Without M^+ in the organic phase [eq. (S2)], $\Delta G^{\circ}_{\text{chem}} = +57.3$ kJ mol^{-1} calculated from eq. (S4) has been reported before.[1] The results in Table S1 indicate that ORR to H_2O_2 in the DCE phase is not favored thermodynamically. However, this step is significantly exergonic in the presence of M^+ in sequence of $Li^+ > Na^+ > K^+$.

Table S1. Calculation of ΔG°_{tot} for overall reaction eq. (S1) or (S2).

Cation	Eqs.	$\Delta^{w}_{DCE}G^{\circ,w\rightarrow DCE}_{H_2O_2}$ / kJ mol^{-1} [a]	$\Delta^{w}_{DCE}G^{\circ,w\rightarrow DCE}_{OH^-}$ / kJ mol^{-1} [b]	ΔG°_{et} / kJ mol^{-1} [c]	$\Delta^{w}_{DCE}G^{\circ,w\rightarrow DCE}_{M^+}$ / kJ mol^{-1} [b]	$\Delta^{w}_{DCE}G^{\circ,w\rightarrow DCE}_{DMFc^+}$ / kJ mol^{-1} [d]	ΔG°_{chem} / kJ mol^{-1}
--	S2, S4	15.4	63.3	151	--	24.1	+53.3
Li^+	S1, S3	15.4	63.3	151	59.8	--	-110.62
Na^+	S1, S3	15.4	63.3	151	58.5	--	-108.02
K^+	S1, S3	15.4	63.3	151	52.3	--	-95.62

[a] The value is assumed the same as for $\Delta^{w}_{DCE}G^{\circ,w\rightarrow DCE}_{H_2O}$, which is calculated based on solubility of water in DCE.[2]

[b] The values are used from literature.[3]

[c] It is calculated from eq. S3 with $\left[E^{\circ}_{O_2/H_2O_2,OH^-}\right]^{DCE}_{SHE}$ = -0.74 V[1] and $\left[E^{\circ}_{DMFc/DMFc^+}\right]^{DCE}_{SHE}$ = 0.04 V. [4]

[d] The value is used from literature.[1]

[1] H. Deng, P. Peljo, T. J. Stockmann, L. Qiao, T. Vainikka, K. Kontturi, M. Opallo, H. H. Girault, *Chem. Commun.* **2014**, *50*, 5554–5557.

[2] a) Z. Samec, *Pure Appl. Chem.* **2004**, *76*, 2147–2180; b) I. Hatay, B. Su, F. Li, M. A. Mendez, T. Khoury, C. P. Gros, J.-M. Barbe, M. Ersoz, Z. Samec, H. H. Girault, *J. Am. Chem. Soc.* **2009**, *131*, 13453–13459;

[3] M. Zhou, S. Gan, L. Zhong, X. Dong, J. Ulstrup, D. Han, L. Niu, *Phys. Chem. Chem. Phys.* **2012**, *14*, 3659–3668.

[4] I. Hatay, B. Su, F. Li, R. Partovi-Nia, H. Vrubel, X. Hu, M. Ersoz, H. H. Girault, *Angew. Chem. Int. Ed.* **2009**, *48*, 5139–5142.
